# Notes and News

**Published:** 1895-02-09

**Authors:** 


### Notes and News.

Collected and Communicated.

### HOSPITAL MEETINGS.

St. Thomas's Hospital.—Public meeting at the Mansion House, on Wednesday, February 13th, at 3 p.m.

City of London Hospital for Diseases of the Chest, Victoria Park.—Annual Court at the Hospital, Thursday, February 14th, at 4 p.m.

Royal National Hospital for Consumption.—General meeting at 34, Craven Street, Charing Cross, on February 14th, at 4.30 p.m.

St. Mark's Hospital for Fistula.—General meeting at the Mansion House, February 14th, at 11 a.m.

London Fever Hospital.—General meeting of Governors at Hotel Victoria, February 15th, at 5 p.m.

In France the hospitals benefit by a tax on betting on horse races. Last year the sum received by the charities from this source amounted to about £10,000.

At Liverpool the scheme to erect a new Northern Hospital on another site is advancing. The new institution will be provided through the munificence of the David Lewis trustees, with the assistance of the corporation of Liverpool.

The latest suggestion of a source of danger in the matter of infection is physicians' fees. The idea emanates from Hungary. It is recommended that doctors receiving fees from infected quarters should place them in a metal receptacle where they could be thoroughly sterilised.

Facing the attendant dangers of fires is not the only unpleasant contingency connected with the fireman's lot. It is reported that the Liverpool Fire Brigade had to go through the ordeal of disinfection and vaccination before they were allowed to return to public or private life after quenching a fire at a small-pox hospital.

Although it is fully decided that a new infirmary for Newcastle is necessary, the difficulty of selecting a site still remains. The favourite spot is held to be one on the town moor, called the Leazes, but the freemen to whom it belongs have hitherto objected; it appears possible, however, that this objection may be removed.

At the Middlesex Hospital (1) Sir Ralph Thompson, K.C.B., has been elected chairman of the Weekly Board vice Lord Sandhurst. (2) Dr. Duncan has ceased to see out-patients (obstetric) on Wednesdays at 1.30, that duty being now performed by Dr. Boxall. (3) Dr. Boxall has ceased to see obstetric out-patients on Fridays at 9 a.m. (4) Dr. R. Douglas Powell visits his wards on Tuesday (instead of Monday) at 1.30.

A largely-attended demonstration in aid of the National Lying-in Hospital—a Roman Catholic institution—was held in Dublin last week. The excellent work done by the institution was ably put before the assemblage. Nevertheless, nearly the whole of the addresses were occupied by religious controversial statements, and a very sore feeling towards the management of the Rotunda Hospital was visible throughout, although the useful work of that institution amongst the Catholic patients was fully admitted.

The important part which our great City Companies play in the support of our medical charities besides other institutions for the public benefit is not sufficiently widely known to be fully appreciated. Praise or blame is more often awarded to individuals than to a component body, yet a City Company is able to be a more permanent giver than the wealthiest of mortals, and is consequently a most valuable factor. As an example of the generosity which emanates from the City, we take the list of donations by the Leathersellers' Company. Last year no less than £1,066 were given to the medical charities alone. The Company maintains a large number of alms-people and pensioners, and has several other benevolent schemes of its own, besides contributing to all forms of charity, and encouraging to a large extent both art and learning. On all these objects the Leathersellers' Company expended £5,338 during 1894.

The full report of the proceedings at the meeting of the Governors of the Radcliffe Infirmary has excited general interest throughout the hospital world. It is believed that the committee will now set to work to re-organise their executive, place the medical administration in the hands of a resident medical superintendent, and the financial and general business in the hands of a strong and experienced secretary. We hope that both these steps will be taken without delay. We would further suggest that it would be a practical step towards uniting the University to the Infirmary, to approach Professor Burdon Sanderson with a view to his joining the committee of management. Steps should also be taken to appoint a successor to the late Dr. Darbyshire, whose physicianship has been left vacant since Dr. Darbyshire's death, in the hope that the new Regius Professor of Physic would take charge of some beds in the infirmary. As we understand that Professor Sanderson is not likely to undertake this work, we would suggest the selection of the strongest man that can be found, for the appointment of honorary physician, so as to increase the weight of medical authority in the institution.

